# Notes and News

**Published:** 1896-08-01

**Authors:** 


### NOTES AND NEWS.

(Collected and Communicated.)

Upwards of £4,000 was secured towards the Middlesex Hospital Convalescent Home by the recent interesting fête held at the hospital.

The Spanish troops in Cuba are severely attacked by the prevailing epidemic of yellow fever. The mortality is large, amounting to 40 to 60 per cent. of the patients.

The charming Rose Fête, held in aid of the North-Eastern Hospital for Children, secured £2,800. The sum of £500 is yet wanted to place the hospital on a satisfactory financial basis.

Already preparations are being made to commemorate the sixtieth year of our Queen's reign. The Victoria Hospital for Children at Chelsea is already arranging for a grand fête and fancy fair in June, 1897.

It is reported that Bournemouth is to receive the gift of a most important new medical institution. The husband of a lady whose life was prolonged for some years by residence in Bournemouth has made an offer to the National Sanatorium there of a new sanatorium costing £50,000 and a beautiful site of 10 acres in Talbot Woods.

The galleries of the Royal Institute of Painters in Water Colours were the scene of a bright and interesting gathering, last Friday, in connection with the distribution of prizes to the students of the Dental Hospital of London. At the distribution the chair was taken by Mr. Morton Swale, the Dean of the schools, and excellent speeches were made by him and Sir J. Crichton Browne. After the formal proceedings were over a pleasant evening was spent, and some excellent music enjoyed.

The Friendly Societies' Convalescent Home at Dover, which has been under alteration, has been reopened by Sir Albert Rollit.

The annual meeting of the Grosvenor Hospital for Women and Children, Vincent Square, S.W., will be held on Friday, July 31, at five p.m., in the new out-patients' department in rear of old hospital.

On Tuesday the Duchess of Connaught laid the foundation-stone of a cottage hospital for Aldershot. The building will be erected on a breezy, open site, and will contain ten beds. At the ceremony, in which the Duke of Connaught took an active part, purses were presented to the Duchess, the collected sum amounting to £800. A donation of 100 guineas was announced from the Empress Eugénie, and the Duchess herself made a donation of twenty-five. The whole amount is to be devoted to the maintenance fund. The town of Aldershot was gay with flags and floral decorations, and the greatest interest in the occasion was universal. The weather was brilliant, and a large company assembled to welcome their Royal Highnesses. The purses were presented by small children who performed their part very prettily.

It has not been generally understood that H.R.H. the Prince of Wales has succeeded Lord Aldenham as president of Guy's Hospital, and that Mr. Lushington, after many years of office, has resigned the post of treasurer to that institution. It is, we understand, practically settled that Mr. Lushington will be succeeded in the office of treasurer by Mr. Cosmo Bonsor, M.P., who has rendered most valuable services to the hospitals of London as a member of the Distribution Committee of the Hospital Sunday Fund. We have little doubt that Guy's will not in future have a resident treasurer, that steps will be taken to place the institution upon a popular basis, and that within the next two years the income of Guy's Hospital will be increased to a sum sufficient to open a new nurses' home, which is badly needed, to remodel the private nursing institution, and to thoroughly equip the whole institution so that Guy's may become once more the completest and best administered medical institution in the country.

The present Lord Mayor, Sir Walter Wilkin, has taken the deepest interest in the Hospital Sunday collections with the view of increasing the amounts raised in the larger churches. At Sir Walter Wilkin's suggestion a letter was addressed to the clergy and ministers asking them to send a special letter to the wealthier members of their congregations soliciting a special contribution for the hospitals. The result has been most satisfactory, and in the case of twenty-nine congregations to whom the letter was sent the average increase from the collections amounts to £30, the total yield showing an increase of nearly £900 in 1896 as compared with 1895. The collections in Paddington at the parish church and St. Michael's both show a falling off this year as compared with last, a fact which is not creditable to the clergy or the wealthier residents in that parish, especially as the collections at St. Stephen's, Westbourne Park, have increased nearly 100 per cent. within the last few years.

